# Dexamethasone Treatment for Covid-19, a Curious Precedent Highlighting a Regulatory Gap

**DOI:** 10.3389/fphar.2020.621934

**Published:** 2020-11-30

**Authors:** Lucia Gozzo, Laura Longo, Daniela Cristina Vitale, Filippo Drago

**Affiliations:** ^1^ Clinical Pharmacology Unit, Regional Pharmacovigilance Centre, University Hospital of Catania, Catania, Italy; ^2^ Department of Biomedical and Biotechnological Sciences, University of Catania, Catania, Italy; ^3^ Centre for Research and Consultancy in HTA and Drug Regulatory Affairs, University of Catania, Catania, Italy

**Keywords:** dexamethasone, COVID19, drug repurposing, off-label, European Medicines Agency

## Introduction

The centralized marketing authorization issued by the European Medicines Agency (EMA) valid in all European Union (EU) Member States has been introduced with the Council Regulation (EEC) No. 2309/93 (Council Regulation, 1993), and improved and amended by the Regulation (EC) No. 726/2004 (Regulation EC, 2004), which introduced the obligation of the procedure for some products, including treatment for viral diseases. Therefore, all the drugs and vaccines developed for Covid-19 must be approved according to this approach to be marketed throughout the EU.

Moreover, in order to deal with the emergency and expedite drug and vaccine development for Covid-19, EMA implemented several rapid procedures in addition to those already provided with the standard “accelerated assessment” (Supplementary Material A1).

Conversely, EMA has no power in pricing and reimbursement decisions, which remain the responsibility of the national competent authorities, due to the heterogeneity of the specific national health system (NHS) organization and financial resources.

According to the Article three of the Regulation (EC) No. 726/2004, application for each marketing authorization shall be submitted by the company to the Agency which will issue an opinion through the Committee for Medicinal Products for Human Use (CHMP). This Committee is solely responsible for releasing the opinions (publicly accessible) on all matters about medicinal products for human use.

Moreover, the Article five of the Regulation provides for the possibility that the Executive Director of the European Medicines Agency (EMA) or the European Commission (EC) or a Member State can request the CHMP to formulate an opinion on issues of particular relevance concerning medicinal products for human use.

Supplementary Material A2 shows EMA opinions issued till October 2020 according to Article five of Regulation 726/2004. Almost 60% of the queries concerned safety issues, in general or in special populations (e.g., elderly, pediatric patients, pregnant women).

More than half of the assessments were required by the Executive Director of the EMA or by EC and about 40% by national authorities.

On July 2020, CHMP started to review the data concerning the use of dexamethasone in patients with Covid-19 (EMA, 2020a) at the request of the EMA Executive Director following a discussion with European experts belonging to the Covid-19 EMA pandemic task force (COVID-ETF).

In the light of the results of the review, the Committee concluded that dexamethasone can be considered a treatment option for patients who require oxygen therapy (including supplemental oxygen and mechanical ventilation; EMA, 2020b).

### Evidence Supporting Dexamethasone Use in Covid-19 and European Positive Opinion

The available data assessed were those of the RECOVERY (Randomised Evaluation of COVid-19 thERapY) study arm, which provided for the use of dexamethasone as add-on therapy to the standard of care of hospitalized patients with Covid-19.

The RECOVERY study (University of Oxford, 2020) is a randomised, controlled, open-label, multicenter (involving 176 National Health Service organizations in the United Kingdom), adaptive trial designed to assess the effects of potential treatments in adults patients hospitalized with Covid-19, receiving invasive or non-invasive ventilation, and those receiving or not oxygen. The study is supported by the National Institute for Health Research-Clinical Research Network (NIHR-CRN), which funds high-quality health and care research in England.

According to the adaptive design, an independent Committee was responsible for the assessment of the interim trial results, which would be made available to the public in case of strong evidence on mortality. Moreover, in this case or if other candidate therapeutics with supporting evidence should be evaluated, the trial arms would have been amended accordingly.

One of the first version of the protocol provided the following arms (RECOVERY, 2020a):

- no additional treatment
- lopinavir-ritonavir
- interferon *β*
- low-dose corticosteroids
- hydroxychloroquine.

In a subsequent version the interferon *β* arm has been deleted and replaced by azithromycin one (RECOVERY, 2020b). In addition, the new protocol allowed a second randomization (no additional treatment vs. tocilizumab) for patients with evidence of hyper-inflammatory state (RECOVERY, 2020c).

Then the trial design was further modified, and eligible patients were allocated simultaneously to no additional treatment vs. convalescent plasma vs. synthetic neutralizing antibodies (RECOVERY, 2020d).

Finally on June 2020, the interim analysis showed important (and opposite) results which led to the withdrawal of three arms (RECOVERY, 2020e):

- Dexamethasone arm due to the demonstration of death reduction by up to one third in hospitalized patients with severe respiratory complications of Covid-19.
- Lopinavir-ritonavir and hydroxychloroquine due to the lack of clinical benefit (RECOVERY Collaborative Group, 2020).

The trial continues randomization to groups receiving azithromycin, tocilizumab, or convalescent plasma.

Overall 6,425 patients (89% with a laboratory-confirmed SARS-CoV-2 infection) were enrolled and randomized to receive either dexamethasone (2,104 patients) or usual care alone (4,321 patients) (RECOVERY Collaborative Group, 2020).

Among randomized patients, 60% required oxygen therapy, 16% invasive mechanical ventilation or extracorporeal membrane oxygenation, and 24% neither.

The primary endpoint was the mortality at 28 days was significantly lower in the dexamethasone group (22.9% death) than in the comparator one (25.7%; rate ratio, 0.83; 95% confidence interval [CI], 0.75 to 0.93; P < 0.001). In particular, the difference between groups was clear for patients receiving invasive mechanical ventilation (29.3 vs. 41.4%; rate ratio, 0.64; 95% CI, 0.51–0.81) and in those receiving supplementary oxygen without invasive mechanical ventilation (23.3 vs. 26.2%; rate ratio, 0.82; 95% CI, 0.72–0.94).

On the contrary, no reduction in the risk of death was obtained with the administration of dexamethasone in patients who were not receiving any respiratory support (17.8 vs. 14.0%; rate ratio, 1.19; 95% CI, 0.91–1.55). Moreover, the duration of hospitalization in the dexamethasone group was shorter than those in the usual care group especially among patients mechanically ventilated at randomization (rate ratio 1.48; 95% CI 1.16, 1.90), or receiving oxygen (rate ratio, 1.15; 95% CI 1.06–1.24), with no benefit in patients not receiving oxygen (rate ratio, 0.96; 95% CI 0.85–1.08).

These results are supported by additional published data, including a meta-analysis conducted by the World Health Organization (WHO), reporting data from seven clinical studies about the use of corticosteroids for the treatment of patients with Covid-19 (WHO Rapid Evidence Appraisal for Covid-19 Therapies (REACT) Working Group, 2020). The analysis included a total of 1703 patients randomized to receive systemic corticosteroids (dexamethasone, hydrocortisone, or methylprednisolone; n = 678) or usual care or placebo (1,025 patients). The primary endpoint was all-cause mortality at 28 days after randomization.

The study results show a reduced risk of death at 28 days among patients randomized to corticosteroids compared with standard of care or placebo [summary OR, 0.66 (95% CI, 0.53–0.82); P < 0.001 based on a fixed-effect meta-analysis].

Therefore, an inexpensive and commonly used steroid is the first drug showing to prevent deaths from Covid-19 (Ledford 2020).

Based on the data described above, EMA endorsed the use of dexamethasone (oral or injectable) in adults and adolescents (from 12 years of age and weighing at least 40 kg) who require supplemental oxygen therapy, at the recommended dose of 6 mg once a day for up to 10 days (EMA, 2020c).

### From European Approval to Patients’ Access

The procedure under the Article five of the Regulation has allowed to recommend an extension of the use of a product already on the market. This is the first time that a new indication is approved through this procedure. Previously the review of the risk-benefit profile has led to the recommendation of use and dosage in special populations, eg for antiviral and anti-tubercular drugs (EMEA, 2009; EMA, 2012).

However, this recommendation does not translate into an automatic update of the Summary of Product Characteristics (SmPC), and the marketing authorization holders can request to add the new therapeutic use to their product’s license by submitting an application to national regulatory authorities or EMA.

To date EMA received an application from Taw Pharma for the authorization of an injectable dexamethasone for treating hospitalized patients with Covid-19 (EMA, 2020d). The application will be evaluated by the CHMP according to an accelerated assessment. This will enable to issue an opinion within the shortest possible time.

**FIGURE 1:**
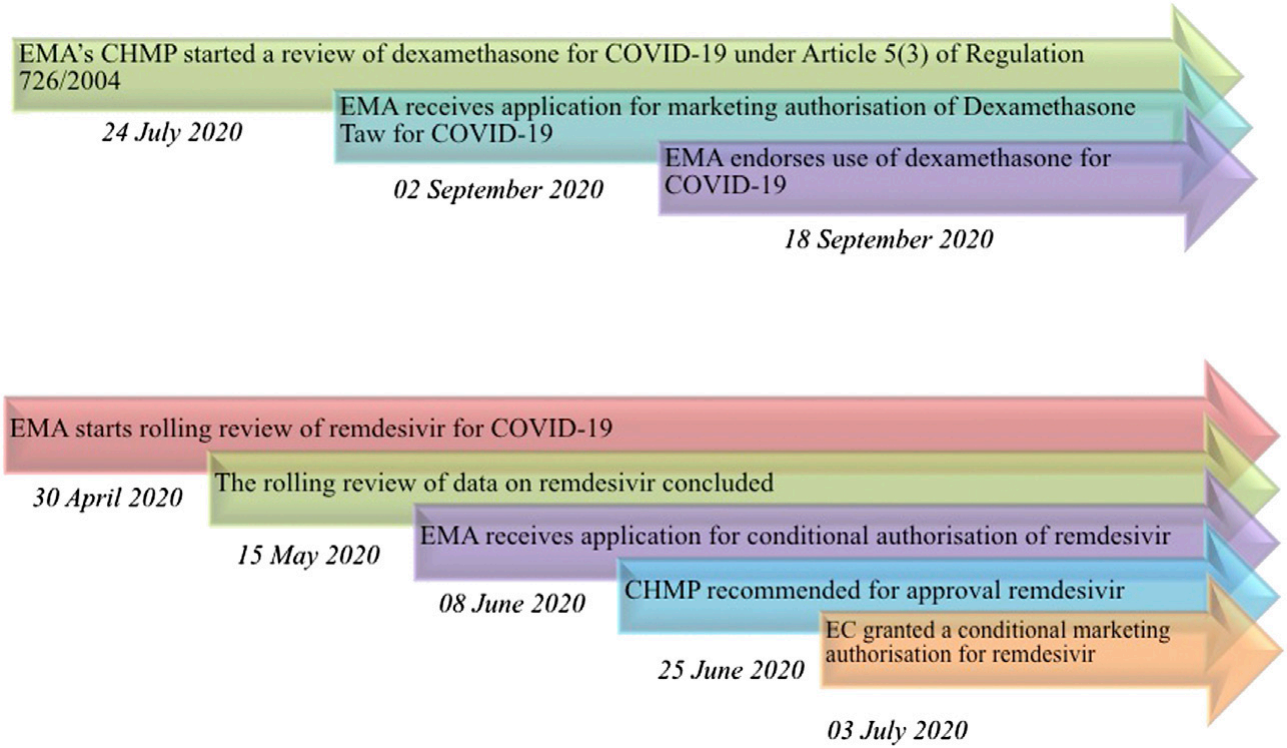
Timeline of drugs' assessment performed by EMA for Covid-19. **(A)** desamethasone; **(B)** remdesivir. *EMA: European Medicines Agency; CHMP: Committee for Medicinal Products for Human Use; EC: European Commission*.

The procedure allowed to deliver the opinion in less than two months (Figure 1A). On the other hand, the rapid assessment of the only drug approved for Covid-19, remdesivir, starting with the emergency procedure of the rolling review, has been completed in almost the same timeframe (Figure 1B). In this case, after the issue of conditional approval, EMA also implemented the *Emergency Support Instrument* (ESI) and subsequently a joint procurement contract in order to guarantee access to the drug throughout Europe (EC, 2020a; EC 2020b).

After CHMP opinion issued about dexamethasone on September 18th^,^ 2020, the European national authorities have not implemented yet any procedure to guarantee access to the drug. In particular, waiting for the conclusive EMA approval following the companies’ application for the final authorization of the new indication, some Member States have the possibility to recognize the nation-wide off-label use according to specific laws. For example, in Italy it is possible to include the off-label use in Covid-19 patients into the List of drugs reimbursed according to Law 648/96, whereas France may start a Temporary recommendation for use (RTU) program. Currently, no such action has been taken.

On October 6th the Italian Medicines Agency (AIFA) published a document with information useful to guide the prescription of corticosteroid in patients with Covid-19 (AIFA, 2020). However, the use has not been officially approved by the same Agency, so the treatment still falls within the scope of the off-label legislation, the so-called “*Di Bella Law”* (Gozzo et al., 2020; Di Bella, 2010), which allows the physicians to perform off-label prescriptions *in individual and exceptional cases*, unless the local Health Director of the hospital formally authorize case by case the use. Anyway, the NHS does not cover the cost of the treatment. The only case in which an off-label use can be reimbursed in Italy is about drugs included in specific lists under the Law 648/1996 (Law 648, 1996). In this context, during the emergency AIFA provisionally endorsed the use and reimbursement of some drugs, such as hydroxychloroquine/cloroquine, lopinavir/ritonavir, and darunavir/cobicistat, despite the non-applicability of the Law 648/1996, subsequently revoked due to the lack of data supporting a favorable risk-benefit profile (Gozzo et al. 2020).

Similarly, two decrees have been published by the French Ministry of Solidarity and Health, governing the prescription, dispensing and administration of hydroxychloroquine for patients with Covid-19:

The Decree n. 2020–314 of March 25, 2020 and n. 2020–337 of March 26, 2020 authorized the prescription, dispensation and administration of hydroxychloroquine and the combination of lopinavir/ritonavir “*under the responsibility of a doctor to patients affected by Covid-19, in the health establishments which take charge of them”, “in particular, for patients with oxygen-demanding pneumonia or organ failure*” (Ministère Des Solidarités Et De La Santé, 2020a; Ministère Des Solidarités Et De La Santé, 2020b).

In this case, these drugs were supplied and paid by health institutions.

Finally, even the French government revoked the decrees that allowed to prescribe hydroxychloroquine, due to the lack of proof of benefit and the health risks (Ministère Des Solidarités Et De La Santé, 2020c).

Given the positive and well-established findings that the drug is currently the only one preventing the mortality of patients, actions to ensure uniform and controlled access to corticosteroids for Covid-19 should be put in place as soon as possible.

The need to fill this regulatory gap is even stronger in light of the recently published interim results of the Solidarity Trial (Dyer, 2020; WHO, 2020; WHO Solidarity trial consortium, 2020).

The study supported by the World Health Organization is one of the largest international randomized trials for Covid-19 treatments, enrolling almost 12,000 patients in over 30 countries and evaluating the effect of drugs on important outcomes such as mortality, need for assisted ventilation and duration of hospitalization.

The preliminary results show little or no effect on these hard endpoints for the four treatments evaluated (remdesivir, hydroxychloroquine, lopinavir/ritonavir and interferon).

These findings confirm that till now only corticosteroids have proven effective in severe and critical Covid-19 patients. It is noteworthy to emphasize that these data come from well-designed clinical trials that it was possible to rapidly start and efficiently conduct despite the emergency status, giving the first specific and evidence-based (although adjustable following future studies) guidance to clinicians on how to manage patients with Covid-19.

## Conclusion

A lot of molecules have been tested in Covid-19 patients, but few positive results have been obtained.

Regulatory authorities react to the emergency adopting a number of measures in order to accelerate drug development and assessment process of available results.

The European procedure regulated by the Article five of the Regulation (EC) No 726/2004 allows to start independently from company interest the assessment of drugs potentially useful for unmet need, such as Covid-19.

However, currently this advantage in terms of time and resource seems to be lost due to the lack of an automatic transferability for prescription in clinical practice, in particular in this emergency situation.

It is dramatically important to rapidly overcome this regulatory gap to made widely available dexamethasone and corticosteroid in general, the only therapeutic option which demonstrated clinical relevant results in Covid-19 so far.

## Author Contributions

LG wrote the first draft of the manuscript. FD checked and revised the draft manuscript. All authors contributed read, revised, and approved the submitted version.

## Conflict of Interest

The authors declare that the research was conducted in the absence of any commercial or financial relationships that could be construed as a potential conflict of interest.
