# Supplementary material for: Dexamethasone Treatment for Covid-19, a Curious Precedent Highlighting a Regulatory Gap
Source: Front Pharmacol. 2020 Nov 30;11:621934. doi: 10.3389/fphar.2020.621934 (PMC7734326; doi:10.3389/fphar.2020.621934)
Supplement: Supplementary file 1 [file datasheet1.docx]

**Appendix 1:** accelerated procedures introduce by EMA to support the development and marketing authorisation of treatments and vaccines for Covid-19 (adapted from <https://www.ema.europa.eu/en/human-regulatory/overview/public-health-threats/coronavirus-disease-covid-19/guidance-medicine-developers-other-stakeholders-covid-19#early-support-for-medicine-and-vaccine-developers-section>)

| **N** | **Procedure** | **Features** |
| --- | --- | --- |
| **1** | ***Rapid scientific advice*** | An ad hoc procedure, which follows the general principles of the standard scientific advice, but with adaptations (no deadline, more flexibility, free of charge, reduced review time) to facilitate development of treatments and vaccines for Covid-19.  Aim: to support prospective evidence planning, to guide on the best method and study design. |
| **2** | ***Rapid agreement of pediatric investigation plans (PIPs) and rapid compliance check*** | EMA review the applications for agreement of PIP for treatments and vaccines for Covid-19 in an expedited manner (no deadline, reduced review time), in order to speed up these products’ development and approval. |
| **3** | ***Rolling review*** | An ad hoc procedure for the public health emergency;  EMA review data of treatments and vaccines as they become available, while the development is still ongoing.  Once the data package is complete, the formal assessment procedure for a marketing authorisation application will take place in a short timeframe. |
| **4** | ***Accelerated assessment*** | Can be considered for medicines and vaccines not undergoing a rolling review. It allows EMA to review the marketing authorisation applications in a shorter timeframe.  Review is reduced to 150 days (from 210 days) or less. |

**Appendix 2:** EMA opinions issued according to Article 5(3) of Regulation 726/2004 (update October 2020; adapted from <https://www.ema.europa.eu/en/human-regulatory/post-authorisation/referral-procedures/article-53-opinions>)

.

| **N** | **Opinion/Assessment report** | **Year** | **Applicant** | **Drug** | **Type** |
| --- | --- | --- | --- | --- | --- |
| 1 | EMEA/CHMP/410051/2006 | 2006 | France | Non-selective non-steroidal anti-inflammatory drugs (NSAIDs) | Safety concern |
| 2 | EMEA/498920/2006 | 2006 | European Commission | /* | Guidelines |
| 3 | EMEA/CHMP/SWP/146166/2007 | 2007 | European Commission | / | Safety concern |
| 4 | EMEA/CHMP/271967/2008 | 2008 | Germany | Heparins | Safety concern |
| 5 | EMEA/CHMP/266022/2008 | 2008 | European Commission | Antidepressants | Safety concern |
| 6 | EMEA/CHMP/590563/2008 | 2008 | United Kingdom | Antipsychotics | Safety concern |
| 7 | EMEA/CHMP/278838/2009 | 2009 | EMEA | Oseltamivir and zanamivir | Risk-benefit profile |
| 8 | EMEA/CHMP/292474/2009 | 2009 | Denmark | Bisphosphonates | Safety concern |
| 9 | EMA/833156/2010 | 2010 | United Kingdom | Peritoneal dialysis solutions | Safety concern |
| 10 | EMA/CHMP/267815/2011 | 2011 | Executive Director of the EMA | Live attenuated viral vaccines | Safety concern |
| 11 | EMA/416998/2011 | 2011 | European Commission° | Celecoxib | Risk-benefit profile |
| 12 | EMA/474456/2011 | 2011 | Germany | Diphtheria and Tetanus toxoid (DT) and Diphtheria/Tetanus toxoid/Pertussis (DTwP) antigen bulks | Safety concern |
| 13 | EMA/CHMP/893625/2011 | 2011 | Italian Medicines Agency (AIFA) | Angiotensin II (type-1) receptor antagonists | Safety concern |
| 14 | EMA/227191/2012 | 2012 | French Medicines Agency (Afssaps) | Anti-tuberculosis medicinal products | Risk-benefit profile |
| 15 | EMA/696137/2012 | 2012 | Medicines and Healthcare Regulatory Agency (MHRA) | Non-Steroidal Anti-Inflammatory Drugs (NSAIDs) | Safety concern |
| 16 | EMA/687578/2012 | 2012 | Executive Director of the EMA | Pandemic vaccines | Safety concern |
| 17 | EMA/741250/2012 | 2012 | Executive Director of the EMA | Protamine sulphate | Quality/Product supply |
| 18 | EMA/227216/2012 | 2012 | Paul-Ehrlich-Institut (PEI) | Human normal immunoglobulin for intravenous administration (IVIg) | Quality/risk-benefit profile |
| 19 | EMA/194668/2013 | 2013 | France | Teicoplanin | Therapeutic equivalence |
| 20 | EMA/474117/2013 | 2013 | / | GLP-1 agonists and DPP-4 inhibitors products | Safety concern |
| 21 | EMA/175205/2014 | 2014 | European Medicines Agency (EMA) | Formulations containing propylene glycol | Safety concern |
| 22 | EMA/CHMP/153652/2015 | 2015 | Executive Director of the EMA | Polymyxin-based products | Quality/risk-benefit profile |
| 23 | EMA/204393/2016 | 2016 | European Medicines Agency (EMA) | Medicinal products under development for the treatment of Ebola | Quality/risk-benefit profile |
| 24 | EMA/421768/2017 | 2017 | German national competent authority (BfArM) | Desloratadine-containing medicinal products | Prescription status |
| 25 | EMA/805330/2018 | 2018 | Executive Director of the EMA | Gentamicin | Safety concern |
| 26 | EMA/245512/2019 | 2019 | Medicines and Healthcare Regulatory Agency (MHRA) | Norethisterone and ethinylestradiol | Safety concern |
| 27 | EMA/194375/2020 | 2020 | Executive Director of the EMA | Direct oral anticoagulants (DOACs) | Risk-benefit profile |
| 28 | EMA/369136/2020 | 2020 | Executive Director of the EMA | Nitrosamine impurities in human medicinal products | Safety concern |
| 29 | EMA/483739/2020 | 2020 | Executive Director of the EMA | Dexamethasone | Risk-benefit profile |
